# Visco-Elastic and Thermal Properties of Microbiologically Synthesized Polyhydroxyalkanoate Plasticized with Triethyl Citrate

**DOI:** 10.3390/polym15132896

**Published:** 2023-06-29

**Authors:** Madara Žiganova, Remo Merijs-Meri, Jānis Zicāns, Ivan Bochkov, Tatjana Ivanova, Armands Vīgants, Enno Ence, Evita Štrausa

**Affiliations:** 1 Institute of Polymer Materials, Faculty of Materials Science and Applied Chemistry, Riga Technical University, 3 Paula Valdena Street, LV-1048 Riga, Latvia; remo.merijs-meri@rtu.lv (R.M.-M.); janis.zicans@rtu.lv (J.Z.); ivans.bockovs@rtu.lv (I.B.); tatjana.ivanova@rtu.lv (T.I.); 2 Laboratory of Bioconversion of Carbohydrates, University of Latvia, 1 Jelgavas Street, LV-1050 Riga, Latvia; armands.vigants@lu.lv; 3 SIA MILZU!, LV-3040 Riga, Latvia; enno.ence@rtu.lv (E.E.); evita.strausa@rtu.lv (E.Š.)

**Keywords:** biopolymers, polyhydroxyalkanoates, plasticization, triethyl citrate, polyhydroxybutyrate

## Abstract

The current research is devoted to the investigation of the plasticization of polyhydroxybutyrate (PHB) and polyhydroxybutyrate-co-hydroxyvalerate (PHBV) with triethyl citrate (TEC). Three different PHB or PHBV-based systems with 10, 20, and 30 wt.% of TEC were prepared by two-roll milling. The effect of TEC on the rheological, thermal, mechanical, and calorimetric properties of the developed compression-molded PHB and PHBV-based systems was determined. It was revealed that the addition of TEC significantly influenced the melting behavior of both polyhydroxyalkanoates (PHA), reducing their melting temperatures and decreasing viscosities. It was also revealed that all the investigated systems demonstrated less than 2% weight loss until 200 °C and rapid degradation did not occur until 240–260 °C in an oxidative environment. Apart from this, a remarkable increase (ca 2.5 times) in ultimate tensile deformation ε_B_ was observed by increasing the amount of TEC in either PHB or PHBV. A concomitant, considerable drop in ultimate strength *σ_B_* and modulus of elasticity *E* was observed. Comparatively, the plasticization efficiency of TEC was greater in the case of PHBV.

## 1. Introduction

Huge amounts of annually generated synthetic plastic waste critically affect the environment. Since 2009 the waste quantity has increased by 24%, whereas in 2019 34.4 kg of plastic waste per person on average was generated in the EU [1]. The environmental issues predominantly are caused by the daily consumption of synthetic polymer products with short life cycles (packaging and disposables). Many products with short life cycles are often mixed in a waste stream making their separation and recycling complicated [2]. Consequently, it is important to develop environmentally sustainable alternatives, primarily for products of short life cycles.

Microbially synthesized polyhydroxyalkanoates (PHAs) are polyesters produced by microorganisms as intracellular granules under nutrient stress. In 1925, Lemognie discovered the simplest form of PHAs, polyhydroxybutyrate (PHB), as a source of energy and carbon storage in microorganisms. Under optimal conditions, above 80% of the dry weight of *Alcaligenis euterophus* is of PHB [3]. Other most studied strains for PHB production are *Ralstonia eutropha* (also known as *Cupriavidus necator*) [4], *Alcaligenes* spp., *Azotobacter* spp. [5], *Bacillus* spp., *Nocardia* spp., *Pseudomonas* spp., and *Rhizobium* spp. [6]. These strains are suitable for the production of not only PHB, but also other members of the PHA family such as poly(3-hydroxyvalerate) (PHV), poly(3-hydroxybutyrate-co-3-hydroxyvalerate) copolymer (PHBV), poly(3-hydroxyoctanoate) (PHO), and poly(3-hydroxynonanoate) (PHN) [4,5,7,8]. Copolymers, such as PHBV, have better stress-strain characteristics than PHB and, therefore, they are more attractive for practical use. A fully biodegradable PHBV has been commercially available since 1990 by the company ICI “Biopol”. However, the share of PHAs is still negligible (1.7% [9]) from the biopolymers global market, which in 2019 was estimated to be 3.8 million tons, in sharp contrast to the fossil-based polymer market of 372 million tons [10]. 

Neat PHB is brittle without modification due to high stereoregularity degree and formation of very large and overlapped spherulites with a high tendency to crack. To resolve this issue PHB is plasticized, which undoubtedly affects its rheological, thermal, and mechanical properties [4,11,12,13]. The most common plasticizers for PHB are (1) esters, such as citrates [14], adipates [13], phthalates [15], diols and (2) polyols, such as poly(ethylene glycol) (PEG) [11] and Laprol [16], respectively, (3) vegetable oils, such as epoxidized soybean oil [16], and (4) terpenes [17]. The influence of some plasticizers on the thermal and mechanical properties of PHB and PHBV is reported in Table 1. Unemura et al. [14] found that TEC is an efficient plasticizer for PHB, gradually changing its mechanical and thermal properties. Requena et al. [11] evaluated the efficiency of adding PEG200, PEG1000, and PEG4000 to PHB by achieving minor improvement in ultimate elongation at break though at decreased stiffness and tensile strength. Scalioni et al. [18] reported that the addition of TEC at a mass fraction of 0.3 resulted in a decrease in the elastic modulus of PHB from 230 MPa to 120 MPa. Rapa et al. [19] obtained samples of PHB/TEC blends displaying maximum elongation at a break of 3.1% by loading of 30% TEC.

Although citrate-plasticized PHB and PHBV systems have been widely investigated, not all the aspects have been completely resolved, for example, thermooxidative behavior at elevated temperatures in the air environment. Apart from this, high price and complicated synthesis of technologically competitive PHA copolymers with high stress-strain characteristics still are one of the main limiting factors for increasing production amounts of the polymer. 

Consequently, in the current research, we have performed a synthesis of PHB using a simple low-cost approach. To reduce brittleness, we have performed melt plasticization of PHB using TEC as a cheap and environmentally friendly plasticizer. To evaluate plasticization efficiency at different TEC contents we have investigated structural, thermogravimetric, rheological, and mechanical properties over a broad temperature range. For comparison, the effect of TEC on the above-mentioned properties of commercially available PHB copolymer (PHBV) with small (1%) hydroxyvalerate content has been investigated.

## 2. Materials and Methods

### 2.1. Materials

PHB homopolymer was obtained from bacteria *Cupriavidus necator NCIMB 11,599* by fermentation on glucose in a fed-batch process with phosphate limitation according to Haas et al. [22]. The PHB was recovered by a modified method of Yang et al. [23]. In short, cells of *Cupriavidus necator* were separated by centrifugation at 4500 rpm for 25 min, and obtained biomass was freeze-dried. PHB was extracted from dry biomass by resuspending in 7% SDS solution and incubating for 20 h at 70 °C. After centrifugation at 8000 rpm for 10 min, the PHB sediment was washed with water four times and freeze-dried.

Poly(3-hydroxybutyrate-co-3-hydroxyvalerate was a commercial product (PHBV, China, Ningbo City, TianAn Biopolymer: ENMAT Y1000) with 1 mol% HV 3-(hydroxyvalerate) content [24]. 

Triethyl citrate (TEC, Burlington, MA, USA, Sigma Aldrich, M_w_ = 276.28 g·mol^—1^, ρ = 1.137 g L^—1^) was used as a plasticizer.

### 2.2. Preparation of Plasticized Systems

**Figure 1:**
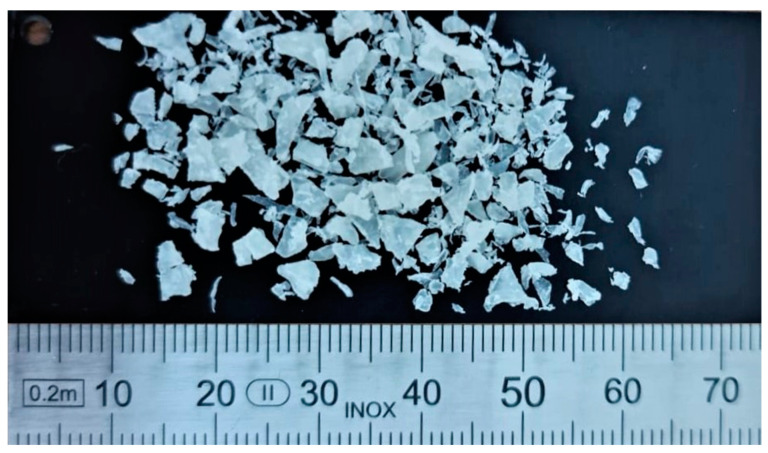
Image of PHBV flakes after milling.

Both biopolymers, before plasticization, were dried at 60 °C in a vacuum oven for 24 h. As shown in Table 2, plasticized systems with TEC weight concentrations of 10%, 20%, and 30% were mixed using a two-roll mill LRM-S-110/3E from Lab Tech Engineering Company Ltd. Mixing time was 3 min and the roll temperatures were 165 °C and 175 °C. Furthermore, the plasticized systems were milled at room temperature and 700 rpm using a Retsch cutting mill SM300 with a 6 mm sieve. The obtained flakes (see Figure 1) with average dimensions of 3 mm × 2 mm were used for manufacturing test specimens using compression molding. Test specimens for mechanical property tests were cut from ~0.5 mm thick plates with dimensions of 60 mm × 100 mm obtained by hot pressing at 190 °C. Samples for oscillatory shear rheology tests were cut from 1 mm thick plates with dimensions of 60 mm × 100 mm, similarly obtained by hot pressing. 

### 2.3. Characterization Methods

#### 2.3.1. Molecular Weight (M_w_)

The viscosity average molecular weight was determined using Ubbelohde viscometer type 1C with diameter of the capillary 0.56 mm (Schott—Instruments GmbH, Mainz, Germany) at 30 °C temperature following the guidelines of ISO 1628. All the samples were dissolved in chloroform to obtain solutions with five different concentrations in the range between 50 mg and 250 mg of PHAs per 100 mL of the solvent. The viscosity average molecular weight for each sample was obtained using the Mark–Houwink equation with K and values of 1.18 × 10^−2^ and 0.780, respectively, as reported elsewhere [4,5,20]:

[η] = k M_w_ ^a^ (1)

where [η] is the intrinsic viscosity of PHAs solutions in chloroform and M_w_ is viscosity average molecular weight.

#### 2.3.2. Fourier Transform Infrared Spectroscopy (FT−IR)

FT-IR spectra were obtained by Thermo Fisher Scientific Nicolet 6700 spectrometer (Thermo Fisher Scientific Inc., Waltham, MA, USA) by Attenuated Total Reflectance (ATR) technique. All the spectra were recorded in the range 650–4000 cm^—1^ with a resolution of 4 cm^—1^.

#### 2.3.3. Thermogravimetric Analysis (TGA)

Therogravimetric properties were analyzed using a Mettler Toledo thermogravimetric analyzer TGA1/SF (Mettler Toledo, Greifensee, Switzerland). Samples of approximately 10 mg were heated from ambient temperature to 600 °C at a heating rate of 10 °C/min under an air atmosphere. The material weight loss was calculated using the original software following the ASTM D3850.

#### 2.3.4. Differential Scanning Calorimetry (DSC)

Melting/crystallization behavior was evaluated using a Mettler Toledo differential scanning calorimeter DSC 1/200W. The specimen of approximately 10 mg was sealed in an aluminum pan and subjected to the following temperature cycles: (1) heating from—50 °C to 200 °C at a rate of 10 °C/min and holding at the corresponding target temperature for 5 min, (2) cooling to 25 °C at a rate of 10 °C/min and holding at the corresponding target temperature for 5 min, followed by (3) second heating from 25 °C to 200 °C at a rate of 10 °C/min. The DSC measurements were performed underneath a nitrogen atmosphere. The degree of crystallinity (χ) was calculated using the following equation:

(2) $$\chi =\frac{\Delta H_{c}}{\Delta H_{m}^{o}\left(1-W\right)}\times 100$$

where $\Delta H_{c}$ is the measured specific melt enthalpy of the compound and $\Delta H_{m}^{o}$ is the melting enthalpy of the 100% crystalline PHB = 146 J/g [12].

#### 2.3.5. Oscillatory Shear Rheology

Discs (ca 1.0 mm (h) × 25 mm Ø) were cut from compression-molded plates of both PHAs and plasticized systems using a die-cutting press and circular die with appropriate dimensions. Complex viscosity η* was measured as a function of angular frequency ω in the oscillatory mode at 190 °C at 1% strain, and within the frequency range of 0.01 Hz to 100 Hz (ω = 0.0628 to 628 rad/s) using a Modular Compact Rheometer SmartPave 102 (Anton Paar GmbH, Graza, Austria—Europe) equipped with 25 mm diameter parallel plate configuration.

#### 2.3.6. Tensile Properties

Tensile stress—strain characteristics were determined at a temperature of 20 °C in accordance with EN ISO 527 using Zwick Roell material testing equipment BDO—FB020TN (Zwick Roell Group, Ulm, Germany) equipped with pneumatic grips. Type 5A test specimens were stretched at a constant deformation speed of 50 mm/min. Demonstrated values represent the averaged results of the measurements performed on 10 test specimens for each type of plasticized system.

#### 2.3.7. Dynamic Mechanical Thermal Analysis (DMTA)

Dynamic mechanical thermal analysis was carried out using METTLER TOLEDO DMA/SDTA861^e^ (METTLER TOLEDO GmbH, Analytical, Schwerzenbach, Switzerland) operating in a tensile mode at 10 N of maximum stress, 10 µm of maximum strain, and a frequency of 1 Hz. Tests were run within a temperature range from −50 °C to +105 °C at a heating rate of 2 °C/min.

## 3. Results

### 3.1. Molecular Weight

Viscosity average molecular weights of PHBV copolymer and PHB homopolymer were calculated from intrinsic viscosity values which were determined using a Ubbelohde type viscometer type 1C with diameter of the capillary 0.56 mm (Schott—Instruments GmbH, Mainz, Germany). The intrinsic viscosity of a polymer is related to its molecular weight, side chain length, and degree of branching. In general, polymers with higher molecular weights and longer side chain lengths have higher intrinsic viscosities, as there is a greater degree of chain entanglement. Intrinsic viscosity also provides information about the conformations of a polymer by reflecting the degree of chain entanglement and intermolecular interactions that occur in a solution.

Linear extrapolation trendlines of PHA viscosity as a function of its solution in chloroform concentration are reported in Supplementary Materials Figure S1. As seen from Figure S1 and Table 3, PHBV shows considerably higher viscosity than PHB, consequently, M_w_ of PHBV is approximately eight times higher than that of PHB. Quagliano et al. [13] have reported that molecular weight, yield, composition, and purity of PHB largely depend on the carbon source and its concentration. For example, it has been observed that by increasing glucose or molasses concentration from 10–50 g/L in the isolated rhizospheric soil samples from the Agronomy Faculty Campus (Buenos Aires, Argentina), the molecular weight of PHB after 24 h of fermentation gradually increased from 55–80 kDa to 300–400 kDa, and 500–700 kDa in the case of glucose and molasses carbon source, respectively [13]. In turn, Luigi-Jules Vandi et al. [24] have reported that the Mw of commercial PHBV with 1 mol% HV 3-(hydroxyvalerate) content, purchased in a powder form from TianAn Biopolymer, China, under the trade name of ENMAT Y1000, usually ranges from 550–650 kDa as analyzed by gel permeation chromatography. The molecular weight of PHB is typically lower than that of PHBV due to differences in their chemical composition and polymerization mechanisms [25,26]. 

### 3.2. Fourier-Transform Infrared Spectroscopy (FT-IR)

**Figure 2:**
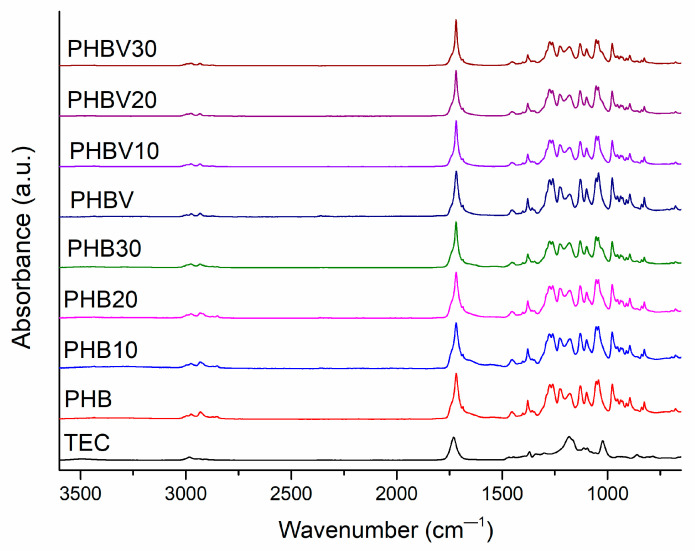
FT-IR spectra of PHAs and their plasticized composites basic functional groups.

**Figure 3:**
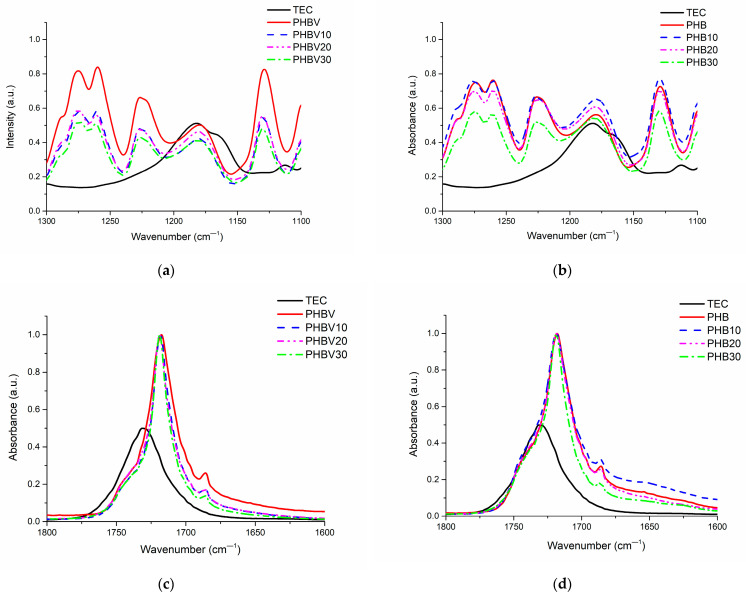
FTIR-ATR spectra of PHBV, PHB, and their plasticized systems within wavenumber range 1725-1740 cm^—1^ (**a**,**b**), and 1150-1240 cm^—1^ (**c**,**d**).

FTIR-ATR spectroscopy was used to assess the structural changes in the plasticized systems after the introduction of TEC. The collected FTIR spectra are shown in Figure 2. In Table 4, representative absorption bands of PHB, PHBV, and TEC are summarized. There is no great difference between the FTIR spectra of PHB and PHBV due to a small amount of HV units in PHBV (1 mol%). The addition of TEC also did not change FTIR spectra dramatically due to structural similarities between the plasticizer and the polymer. The appearance of no new peaks in the FTIR spectra of the plasticized systems also confirms no chemical interaction between TEC and PHB or PHBV. The greatest changes after the addition of TEC have been observed in the carbonyl absorption region; while for TEC, this peak is shifted to the direction of longer wavelengths in comparison to those of PHB and PHBV. However, several bands attributed to C–O–C groups’ asymmetric stretching (1180 cm^−1^ and 1181 cm^−1^ for PHB and PHBV, respectively), C–O–C groups’ symmetric stretching (1130 cm^−1^ and 1129 cm^−1^ for PHB and PHBV, respectively), C-O groups’ vibrations (1226 cm^−1^ and 1274 cm^−1^), and also –C=O groups’ vibrations have been previously related to the ratio of amorphous and crystalline parts of PHAs [27]. Therefore, the main attention was devoted to the assessment of the changes in the crystalline structure of PHB and PHBV after plasticization with TEC. In the current case, respective bands are not significantly shifted due to the addition of TEC. However, the intensity of the bands at 1181 cm^−1^, 1129 cm^−1^, 1226 cm^−1^, and 1274 cm^−1^ of PHBV decreased to a greater extent in comparison to that of the TEC plasticized PHB (see Figure 3). This may be indicative of a larger influence of TEC in the crystalline structure of PHBV in comparison to that of PHB, resulting in more effective plasticization of the copolymer along with addition of TEC. 

### 3.3. Thermal Gravimetric Analysis (TGA)

**Figure 4:**
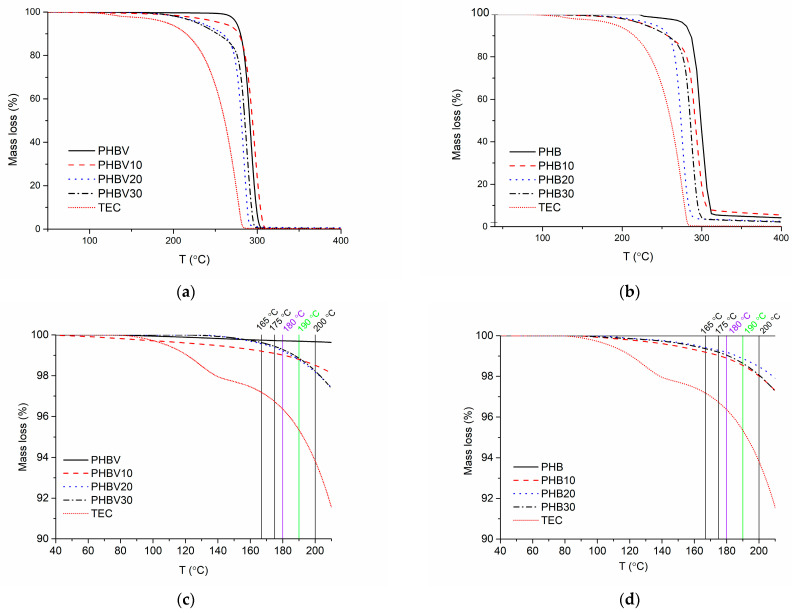
TGA thermograms of TEC plasticized PHBV and PHB systems within the full temperature range (**a**,**b**, respectively) and within the temperature range of 40 °C and 200 °C (**c**,**d**, respectively).

Although many research groups have investigated the thermal behavior of PHAs by TGA, only a few of these investigations have been performed in an oxidative environment, disregarding the fact that even in a closed system, such as an extruder barrel, there is a certain amount of dissolved oxygen [32]. Consequently, TGA tests in the current research have been performed in an oxidative environment. The TGA thermograms of the investigated PHB- and PHBV-based systems are shown in Figure 4. The thermal stability of neat PHB is higher than that of PHBV, which could be explained by the lower deactivation energy of the latter (177 kJ/mol and 136 kJ/mol, respectively) as reported by Yun Chen et al. [27]. As expected, the addition of TEC, which has lower thermal stability, decreased the thermal resistance of the investigated plasticized systems. By increasing the content of TEC, the onset thermal degradation temperature *T_on_* decreases. A relatively larger decrease in *T_on_* is the case for PHB-based systems resulting in the fact that both plasticized systems with 30 wt.% of TEC show almost identical *T_on_.* However, the slope of TGA curves within the main mass loss region for the plasticized systems decreased, testifying that TEC contributes to the formation of the gas-impermeable char layer, reducing the diffusion of oxygen to the zone of burning and decreasing the combustion rate. It should, however, be mentioned that there is negligible mass loss (less than 1%) of the investigated systems if heated up to 190 °C, which was the processing temperature of the investigated systems. In spite of this slight mass loss, the decrease in the molecular weight of PHAs during 30 min of isothermal heating at 180 °C is more than 20% [33], which repeatedly testifies that the processing of PHAs base systems should be performed at possibly low temperatures and short cycle times in Table 5. 

### 3.4. Differential Scanning Calorimetry (DSC)

**Figure 5:**
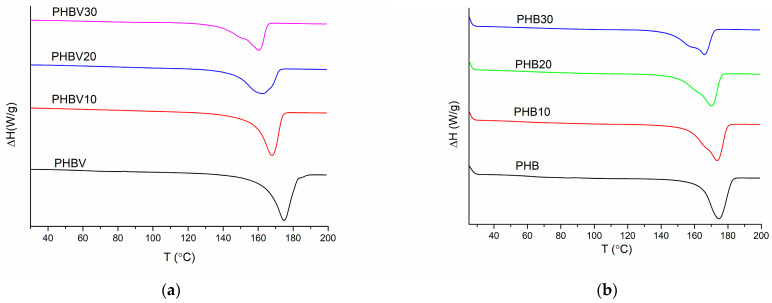
First heating DSC thermograms of the plasticized systems based on PHBV (**a**) and PHB (**b**).

DSC thermograms of the first heating run of all the investigated systems are summarized in Figure 5, whereas the main calorimetric data of the thermograms are given in Table 6. DSC thermograms of the subsequent cooling and second heating runs of PHB, PHBV, and their plasticized systems are reported in the Supplementary Materials Figures S2–S5, whereas the main calorimetric data are summarized in Table 7 for the cooling run and Table 8 for the second heating run. 

It is known that the crystallization of PHAs is affected by nucleation acts and spherulite growth dynamics, which often results in the formation of multimodal exothermic peaks due to the irregular release of heat [34,35]. Consequently, multimodal melting behavior is observed for the investigated PHBV in the first heating run demonstrating one expressed major melting peak at 175 °C, which overlaps with a minor melting peak at 185 °C. The presence of double peaks in melting endotherms is generally explained by two mechanisms: (1) double lamellar thickness population model [36] and (2) melting and recrystallization model [37]. Most probably, the major melting peak of PHBV is attributed to the melting of initially present crystalline structures, which tend to recrystallize into thicker more perfect lamellas. Due to the low co-monomer content, it is believed that the melting peak of HV moieties is overlapped with the melting of dominating HB moieties. This results in a broader melting interval of PHBV in comparison to PHB. During the cooling run, single melting peak of PHBV is observed around 83 °C. In the case of the second run, the bimodal melting behavior of PHBV is observed, whereas the melting peaks are shifted to lower temperatures, which may be because of less crystallization time for the PHA sample, as previously observed by Yun Chen et al. [27]. Similar trends may also be observed from PHB scans, whereas the observed melting/crystallization peak temperatures are somewhat higher in comparison to those of PHBV. 

If TEC is added, the melting endotherm of PHBV systems shifts to the left side resulting in lower peak temperatures of melting of the polymers’ crystalline fractions. It is also worth noting that the addition of TEC, even at its lowest amount (10 wt.%), contributes to the development of multimodal melting behavior, i.e., by increasing TEC concentration, the lower temperature melting peak becomes more separate. This testifies that TEC influences the nucleation process of PHBV. In a similar way, the crystallization peak temperature of the plasticized PHA compositions decreases by TEC addition, and in the case of PHBV-based systems crystallization occurs in a broader range in comparison to PHB/TEC. 

As it is demonstrated in Table 8, controlled crystallization of the investigated systems at the rate of 10 °C/min in the DSC cell, initiated the development of frozen structures, resulting in the appearance of a cold crystallization peak during the second heating run. It may be observed that cold crystallization peak temperature T_cc_ is lowered by the addition of TEC, whereas cold crystallization enthalpy is only slightly affected. Concomitant, cold crystallization affects the initial crystallinity of PHB or PHBV crystalline fraction not more than by a couple of percent. In general, the crystallinity of PHBV is somewhat higher than that of PHB. This is not common behavior; however, it can be explained by a greater amount of crystallizable fractions due to the higher molecular weight of the copolymer. The addition of plasticizer is known to reduce the crystallinity of polymers due to the penetration of plasticizer between polymer macromolecules by reducing intermolecular interaction strength. The observed increase in crystallinity most probably is related to the plasticizer acting as a nucleating agent, promoting the growth of new crystallites or facilitating the aggregation of existing crystallites. Similar behavior has been observed by Jost et al. for a number of different plasticizers including TEC already at small concentrations (5%) [38]. 

### 3.5. Oscillatory Shear Rheology

**Figure 6:**
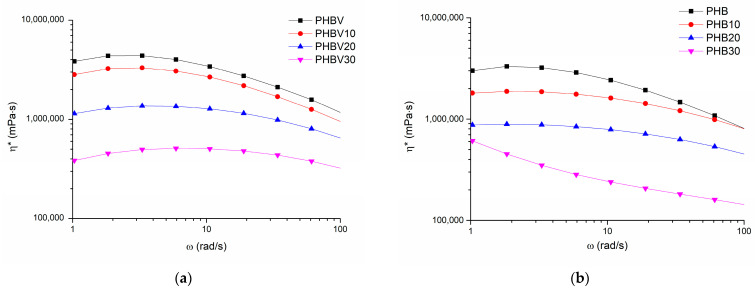
Complex viscosity (η*) versus angular velocity (ω) plots for PHBV (**a**), PHB (**b**), and their plasticized systems (190 °C).

By considering that offset melting temperatures of the investigated PHA systems were within the interval between 168 and 192 °C, oscillatory shear rheology tests were made at 190 °C, close to the highest T_offset_ value. This temperature was also used during the compression molding of the investigated plasticized systems. It has been determined that by increasing shear rates, the complex viscosity η* values of neat PHBV, and PHB as well as the TEC plasticized systems decrease demonstrating shear thinning behavior typical for non-Newtonian fluids (see Figure 6). As already expected, η* of neat PHBV at low angular velocity values ω is higher in comparison to PHB, which is determined evidently by its higher molecular mass. However, at high ω values η* of PHBV becomes smaller than that of PHB, which is explained by the lower thermal stability of PHBV and easier disruption of molecular entanglements due to greater mobility of macromolecular chain of the copolymer caused by valerate moieties. Thus, one may conclude that PHBV is more sensitive to shear stresses than PHB. The addition of TEC decreases conformational rigidity, lowers viscosity, and, hence, eases the processability of the plasticized systems by reducing the intermolecular interactions and disrupting the crystalline structure of the polymers. A relatively smaller decrease in η* for the systems with 10 wt.% of TEC is because the plasticizer molecules may not beefficiently adsorbed between PHBV or PHB chains, leading to a less pronounced reduction in viscosity compared to the systems with higher TEC concentrations.

**Figure 7:**
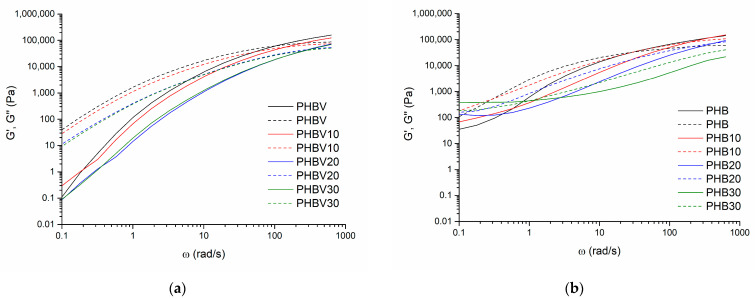
Storage (G″) and loss (G′) modules versus angular velocity (ω) plots for the plasticized systems based on PHBV (**a**), and PHB (**b**) (190 °C).

Besides it has been observed that at the beginning of the oscillatory test (the highest angular velocity value) storage modulus G′ exceeds the loss modulus G″. For example, at an angular frequency of 628 rad/s G′ and G” values are 158 kPa and 87 kPa for neat PHBV and 143 kPa and 59 kPa for neat PHB, respectively. The modulus cross-over point is reached at 100 rad/s for PHBV (60 kPa) and 30 rad/s for PHB (30 kPa), after which G″ starts to dominate over G′. At the lowest angular frequency (0.1 rad/s) respective G′ and G″ values are 0.11 Pa and 40 Pa for PHBV, and 35 Pa and 108 Pa for PHB, testifying that the copolymer has higher shear stress sensitivity. As demonstrated in Figure 7, G′ and G″ of the investigated systems decrease by increasing the TEC content in the polymer composition, especially in the case of plasticized PHBV, following the same trend as matrix polymers. 

### 3.6. Tensile Properties

**Figure 8:**
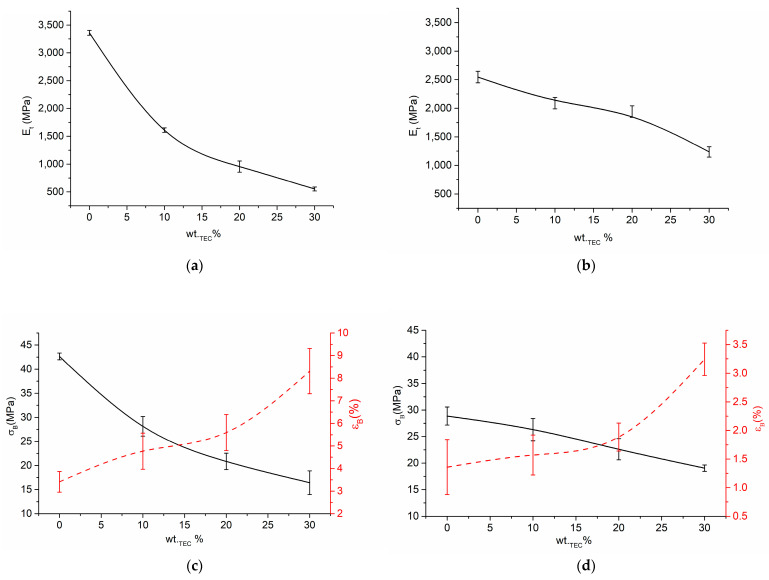
Young’s modulus E (**a**,**b**), stress at break σ_B_ (**c**,**d**), and ultimate deformation ɛ_B_ (**c**,**d**), of the PHBV (**a**,**c**), and PHB (**b**,**d**) based systems.

As seen in Figure 8a,b, by increasing the TEC content up to 30 wt.%, the modulus of elasticity E of the plasticized systems experiences a nearly twofold decrease from 2546 MPa to 1236 MPa and from 3559 MPa to 712 MPa for PHB- and PHBV-based systems, respectively. This indicates that the addition of structurally bulky TEC considerably affects the rigidity of the polymer matrix, especially in the case of PHBV. Consequently, although E of neat PHBV, mainly due to its higher molecular weight, is ca 1.3 times higher than that of neat PHB, after plasticization with 30 wt.% of TEC E of PHBV-based system becomes 2.2 times lower than that of its PHB-based counterpart. Similarly, the addition of TEC has also led to a considerable decrement of stress at break *σ_B_* of all the plasticized systems, especially in the case PHBV-based systems. Thus, due to plasticization with 30 wt.% of TEC *σ_B_* of PHBV- and PHB-based systems decrease by 60% and 40%, respectively. This suggests that PHBV is more efficiently plasticized by TEC in comparison to PHB. Hence, plasticized PHBV-based systems demonstrate 2.5 times larger ultimate elongation values in comparison to PHB-based systems. Disregarding this, ultimate deformation ɛ_B_ of PHB and PHBV due to plasticization increases to a similar extent, i.e., approximately 2.5 times at the maximum TEC concentration. 

### 3.7. Dynamic Mechanical Analysis (DMA) 

**Figure 9:**
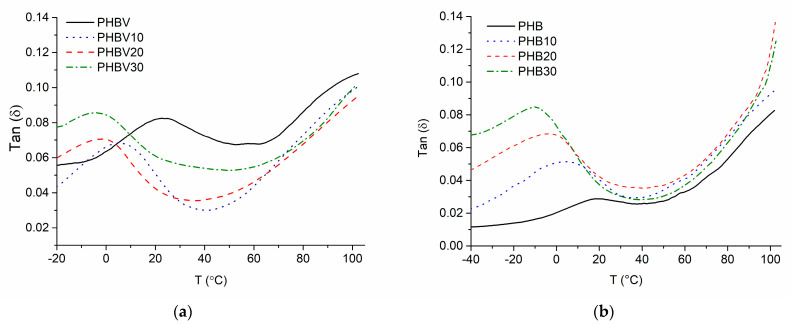
tan δ-(T) relationships of the plasticized systems based on PHBV (**a**) and PHB (**b**).

**Figure 10:**
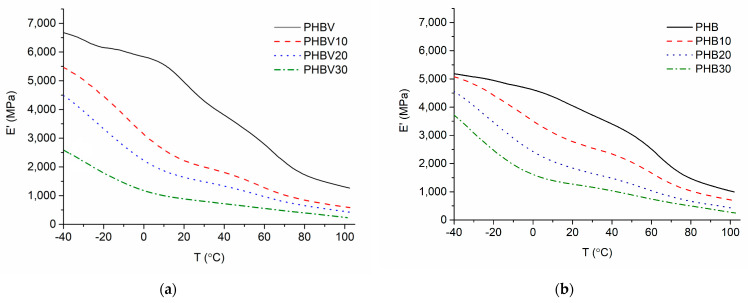
E′(T) relationships of the plasticized systems based on PHBV (**a**), and PHB (**b**).

Loss factor tan δ and storage modulus E′ versus temperature T relationships of the investigated plasticized systems are shown in Figure 9 and Figure 10. The tanδ(T) relationships demonstrate well-expressed relaxation region within temperature intervals −10 °C–+55 °C with maxima at 22 °C for PHBV and between −10 °C and +40 °C with maxima at 19 °C for PHB. This relaxation is associated with glass transition in the amorphous phases of PHB or PHBV. The breadth of this relaxation region is associated with the presence of crystalline fraction in both polymers as previously stated by Scandola et al. [39]. As shown in Table 9, the addition of TEC causes a considerable negative shift in glass transition maxima by 26 °C for both PHBV plasticized with 30 wt.% of TEC and its PHB-based counterpart. This is because the plasticizer causes the weakening of intermolecular forces that contribute to the stiffness of the material. 

Apart from the T_g_ peak, another well-expressed intensity is observed in the tanδ(T) relationships with onset at ca 63 °C and ca 45 °C for PHBV and PHB, respectively. This intensity may be related to the beginning of the crystal–crystal slippage occurring in semicrystalline polymers just below melting as stated by Madbouly et al. [40] and McDonald et al. [6] who observed high-temperature relaxation of PHB at about 110 °C. This transition may also be related to the α′ relaxation of the amorphous–crystalline interphase [27]. The addition of TEC promoted this relaxation process to occur at somewhat lower temperatures, especially in the case of PHBV-based systems, confirming that TEC affects the structure of PHBV to a greater extent in comparison to PHB. 

**Figure 11:**
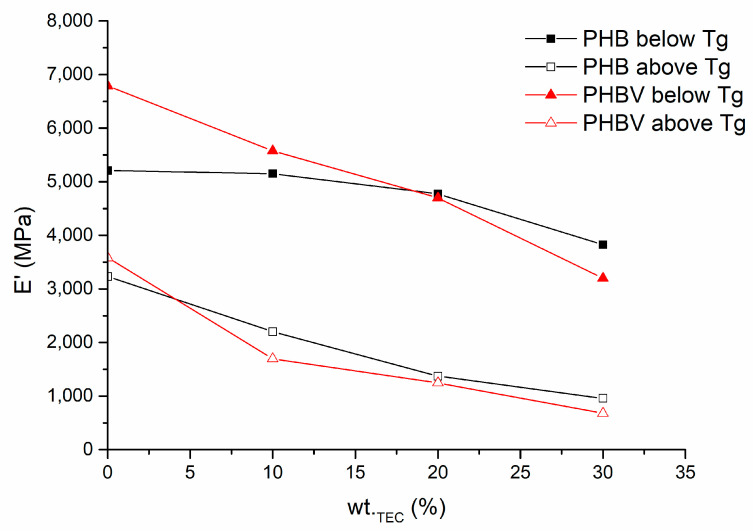
E′ of plasticized systems based on PHBV and PHB before and after T_g_.

In correspondence with tan δ data and trends in tensile properties of the investigated PHA-based systems, storage modulus temperature relationships E′(T) are shifted to the direction of lower temperatures and lower modulus values by increasing TEC content in the plasticized system. As already expected, larger E′ changes have been observed for PHBV-based systems. Figure 11 depicts the E′ change of PHBV and PHB plasticized systems below and above the glass transition region (−45 °C and +45 °C respectively). As expected, below T_g_ the change of E′ of PHBV as a result of TEC addition up to 30 wt.%, is around 45%, whereas the change of the counterpart PHB-based system is only 26%. However, if the temperature is raised above T_g_, the decrement of E′ is much greater, i.e., by ca 70% and 80% for PHB and PHBV-based systems, respectively. Consequently, the TEC addition drop of E′ of PHBV-based systems are more intensive, similarly as it was observed in the case of tensile tests.

## 4. Conclusions

In this research, the efficiency of the plasticization of PHB and PHBV with TEC (10, 20, and 30 wt.%) as an environmentally friendly plasticizer is demonstrated. The following results have been obtained due to plasticization with TEC:

(1) Considerable thermooxidative degradation in the air of the investigated plasticized systems does not occur until 240–260 °C, while the minimum onset thermal degradation temperature is 264 °C;
(2) The rate of thermooxidative degradation of the plasticized systems is decreased to a certain extent due to the contribution of TEC in the building of the gas-impermeable char layer;
(3) Increased shear forces cause decrement of melt viscosity as well as storage and loss modules of both PHB and especially PHB- based systems due to lower activation energy of the latter and weakened interaction between the polymer chains because of plasticization;
(4) The melting range of the plasticized systems is considerably decreased (by ca 10 °C at the maximum peak value), thus relieving the processability of the investigated systems;
(5) Ultimate elongation ε_B_ values of the investigated plasticized systems increase on average 2.5 times by increasing TEC content, reaching values as high as 9% (for PHBV-based systems);
(6) Modulus of elasticity E as well as tensile strength *σ_B_* values experience certain decrements, especially for PHBV-based systems above glass transition temperature T_g_.

Consequently, plasticized low molecular PHB has improved use potential due to reduced brittleness, making it similar to the commercial PHBV in respect to ultimate elongation value. Apart from this, the possibility to process plasticized PHB and plasticized PHBV at somewhat lower temperatures potentially reduces the potential of thermooxidative decomposition of the polymers during melt processing; thus, making them more suitable for the manufacturing of environmentally sound packaging, which is the expected target market of the investigated PHA compositions. To achieve this aim, it is expected to investigate the long-term stability of the developed composites under the influence of different factors of the external environment. It is also expected to assess further modification potential of the developed plasticized systems by using agricultural residues.

## Figures and Tables

**Table 1:** Selected properties of some plasticized PHB systems.

PHAs	TEC(wt. Parts)	Plasticizer	T_m_ (°C)	X (%)	ε_B_ (%)	Reference
PHB	0	Epoxidized linseed oil (ELO)	175	52	9.7	[20]
	0.05		173	47	12.7	
	0.1		171	46	13.6	
	0.05	Epoxidized soybean oil (ESBO)	173	47	9.2	
	0.1		172	48	8.9	
PHB	0	Triethyl citrate (TEC)	180	81	5.8 ± 0.6	[14]
	0.1		173	71	5.6 ± 0.4	
	0.2		171	62	7.4 ± 0.9	
	0.3		162	53	6.9 ± 1.6	
PHB	0	Dioctyl (o-)phthalate (DOS)	169	56 *	2.5 ± 0.5	[15]
	0.25		164	54 *	3.9 ± 0.3	
	0.3		163	57 *	4.3 ± 0.6	
	0.35		165	50 *	5.4 ± 0.9	
	0.4		165	57 *	5.2 ± 0.6	
	0.5		165	51 *	-	
	0	Acetyl tributyl citrate (ATBC)	169	56 *	2.5 ± 0.5	
	0.1		163	61 *	6.1 ± 0.8	
	0.2		160	58 *	8.5 ± 0.9	
	0.25		158	60 *	8.8 ± 0.9	
	0.3		157	59 *	9.7 ± 0.7	
PHBV	0	Biodegradable oligomeric polyester based on lactic acid, adipic acid, and 1,2-propanediol at a molar ratio of 20:40:40 (PLAP)	174	53	8 ± 0.4	[21]
	0.1		173	55	8.2 ± 0.4	
	0.2		170	56	8.1 ± 0.4	
	0.3		174	61	6.6 ± 0.5	

* Crystallinity calculated from DSC data by assuming the enthalpy of 100% crystalline PHB = 146 J/g as reported by [12].

**Table 2:** Codes and the composition of the plasticized PHB and PHBV systems.

Code	PHBV (wt.%)	PHB (wt.%)	TEC (wt.%)
PHBV	100	—	0
PHBV10	90	—	10
PHBV20	80	—	20
PHBV30	70	—	30
PHB	—	100	0
PHB10	—	90	10
PHB20	—	80	20
PHB30	—	70	30

**Table 3:** Molecular weight and instinct viscosity of PHAs.

Sample Code	M_w_ (kDa)	η (Pa·s)
PHBV	540	0.69
PHB	66	3.86

**Table 4:** Representative FT-IR absorption intensities of PHAs [28,29,30,31].

Mode of Molecular Vibration	PHB		PHBV		TEC	
	Current Research	Reference	Current Research	Reference	Current Research	Reference
C–C backbone stretching	978	—	977	977 [28]	—	—
O−C−C stretching	1043/1054	—	1044/1054	1054 [28]	1023	—
O–C–C asymmetric stretching	1100	1000–1300 [29]	1099	1099 [28]	1096 1113	1097 [31] 1114 [31]
C–O–C symmetric stretching	1130		1129	1129 [28]	—	1050-1300 [31]
C–O–C asymmetric stretching	1180		1181	1179 [28]	1182	
C–O symmetric stretching	1226		1226	1226 [28]	—	
C–O symmetric stretching of aliphatic esters	1260		1261	1261 [28]	—	
C–O symmetric stretching	1274		1274	1275 [28]	—	
C–H symmetric bending of methyl (-CH_3_) groups	1379	1377 [29]	1379	1379 [28]	1370	1373 [31]
C–H asymmetric stretching and bending vibrations of methyl (-CH_3_) and methylene (-CH_2_-) groups	1453	1452 [29]	1452	1452 [28]	—	—
C=O stretching of ester groups	1718	1727 [29]	1718	1720 [28] 1722 [30]	1730	1735 [31]
—CH_3_ symmetric stretching	2851/2873	—	2851/2873	2881 [30]	—	—
—CH_2_ symmetric stretching	2390	—	2932	2933 [28] 2925/2945 [30]	—	—
C−H asymmetric vibration of methyl (-CH_3_) groups	2975	2927/2969 [29]	2976	2975 [28]	2982	2983 [31]
Terminal –OH group	3434	3434 [29]	3435	3434 [28]	3484	3502 [31]

**Table 5:** Percent mass loss temperatures of PHBV, PHB, and its plasticized systems.

Sample Code	Residual Mass at Fixed Temperature, wt.%			T_on_, °C	Percent Mass Loss Temperatures, °C		
	180 °C	190 °C	200 °C		T_1%_	T_5%_	T_deg_
PHB	100	100	100	288	227	279	298
PHB10	100	99	99	280	202	246	258
PHB20	100	99	98	264	185	241	274
PHB30	100	99	98	275	181	229	286
PHBV	100	100	100	283	259	276	295
PHBV10	99	99	99	285	208	257	299
PHBV20	99	99	98	279	184	234	283
PHBV30	99	99	98	275	187	228	287
TEC	96	95	94	276	120	190	276

**Table 6:** Results of the first heating run of neat PHBV, PHB, and TEC plasticized systems.

Sample Code	wt. %	1st Heating Run						
		χ	ΔH_m_	T_m_^1^	T_m_^2^	T_onset_	T_offset_	ΔT
		(%)	(J/g)	(°C)	(°C)	(°C)	(°C)	(°C)
PHB	0	53	77	-	174	136	183	47
PHB10TEC	10	58	76	165 *	172	133	181	48
PHB20TEC	20	59	69	160 *	169	127	177	50
PHB30TEC	30	60	61	157 *	166	122	173	51
PHBV	0	64	94	-	175/185 *	135	192	57
PHBV10%TEC	10	59	78	-	168	118	177	59
PHBV20%TEC	20	57	66	-	163	114	174	60
PHBV30%TEC	30	59	60	151*	161	106	168	62

* Relates to inflection point.

**Table 7:** Results of the cooling run of neat PHBV, PHB, and TEC plasticized systems.

Sample Code	wt.%	Cooling Run					
		χ (%)	ΔH_m_ (J/g)	T_m_^1^ (°C)	T_onset_ (°C)	T_offset_ (°C)	ΔT (°C)
PHB	0	45	66	91	67	112	45
PHB10TEC	10	44	58	71	51	100	49
PHB20TEC	20	47	55	75	49	100	51
PHB30TEC	30	45	46	69	43	96	53
PHBV	0	46	68	83	55	108	53
PHBV10%TEC	10	47	61	81	52	106	54
PHBV20%TEC	20	44	51	75	44	99	55
PHBV30%TEC	30	45	46	71	31	98	67

**Table 8:** Results of the second heating run of neat PHBV, PHB, and TEC plasticized systems.

Sample Code	wt.%	2nd Heating Run									
		χ_cc_ (%)	ΔH_cc_ (J/g)	T_cc_ (°C)	χ * (%)	ΔH_m_ (J/g)	T_m_^1^ (°C)	T_m_^2^ (°C)	T_onset_ (°C)	T_offset_ (°C)	ΔT (°C)
PHB	0	3	4	99	54 (51)	78	169	173	139	183	44
PHB10TEC	10	2	2	89	59 (57)	77	156	167	131	176	45
PHB20TEC	20	3	4	89	59 (56)	69	154	166	129	175	46
PHB30TEC	30	4	4	83	62 (58)	63	157	165	127	174	47
PHBV	0	5	8	95	61 (56)	90	167	172	128	185	57
PHBV10%TEC	10	3	4	94	62 (58)	81	162	170	118	176	58
PHBV20%TEC	20	5	6	91	63 (58)	74	154	165	117	175	58
PHBV30%TEC	30	4	5	90	62 (58)	64	148	162	112	172	60

* Value in the brackets reflects the initial crystallinity of the PHB or PHBV by considering the cold crystallization effect.

**Table 9:** Tg from tan δ peak maximum.

Sample	T_g_, °C	Sample	T_g_, °C
PHBV	22	PHB	19
PHBV10	5	PHB10	12
PHBV20	−1	PHB20	−5
PHBV30	−4	PHB30	−7

## Data Availability

The data presented in this study are available on request from the corresponding author.

## Supplementary Materials

The following supporting information can be downloaded at: https://www.mdpi.com/article/10.3390/polym15132896/s1, Figure S1: Viscosity of PHB and PHBV versus solution concentration; Figure S2: Thermograms of DSC cooling run of the PHBV-based systems; Figure S3: Thermograms of DSC cooling run of the PHB based systems; Figure S4: Thermograms of DSC 2nd heating run of the PHBV-based systems; Figure S5: Thermograms of DSC 2nd heating run of the PHB-based systems.
